# There are significant differences among artificial intelligence large language models when answering scientific questions

**DOI:** 10.3389/frai.2025.1664303

**Published:** 2025-10-09

**Authors:** Francisco Javier Álvarez-Martínez, Luis Esteban, Lucas Frungillo, Estefanía Butassi, Alessandro Zambon, María Herranz-López, Mario Aranda, Federica Pollastro, Anne Sylvie Tixier, Jose V. Garcia-Perez, David Arráez-Román, Andrew Ross, Pedro Mena, Ru Angelie Edrada-Ebel, James Lyng, Vicente Micol, Fernando Borrás-Rocher, Enrique Barrajón-Catalán

**Affiliations:** ^1^Institute of Research, Development and Innovation in Health Biotechnology of Elche (IDiBE), Universitas Miguel Hernández (UMH), Elche, Spain; ^2^CEDER-CIEMAT, Centro de Desarrollo de Energías Renovables—Centro de Investigaciones Energéticas Medioambientales y Tecnológicas, Lubia, Spain; ^3^School of Biological Sciences, Institute of Molecular Plant Sciences, University of Edinburgh, Edinburgh, United Kingdom; ^4^Área Farmacognosia, Facultad de Ciencias Bioquímicas y Farmacéuticas, Universidad Nacional de Rosario, Rosario, Argentina; ^5^Department of Civil, Chemical, Environmental, and Materials Engineering, University of Bologna, Bologna, Italy; ^6^Departamento de Química y Farmacia, Pontificia Universidad Católica de Chile, Macul, Santiago, Chile; ^7^Department of Pharmaceutical Sciences, University of Eastern Piedmont, Novara, Italy; ^8^Avignon Université, INRAE, UMR SQPOV, Avignon, France; ^9^Instituto de Ingeniería de Alimentos-FoodUPV, Universitat Politécnica de València, Valencia, Spain; ^10^Department of Analytical Chemistry, University of Granada, Granada, Spain; ^11^School of Chemical and Process Engineering, University of Leeds, Leeds, United Kingdom; ^12^Human Nutrition Unit, Department of Food and Drug, University of Parma, Parma, Italy; ^13^Strathclyde Institute of Pharmacy and Biomedical Sciences, University of Strathclyde, Glasgow, United Kingdom; ^14^UCD School of Agriculture and Food Science, University College Dublin, Dublin, Ireland; ^15^Departamento de Estadística, Matemáticas e Informática, Universidad Miguel Hernández, Elche, Alicante, Spain

**Keywords:** large language models, artificial intelligence, scientific evaluation, prompt engineering, retrieval-augmented generation

## Abstract

**Introduction:**

This study investigates the efficacy of large language models (LLMs) for generating accurate scientific responses through a comparative evaluation of five prominent free models: Claude 3.5 Sonnet, Gemini, ChatGPT 4o, Mistral Large 2, and Llama 3.1 70B.

**Methods:**

Sixteen expert scientific reviewers assessed these models in terms of depth, accuracy, relevance, and clarity.

**Results:**

Claude 3.5 Sonnet emerged as the highest scoring model, followed by Gemini, with notable variability among the other models. Additionally, retrieval-augmented generation (RAG) techniques were applied to improve LLM performance, and prompts were refined to improve answers. The results indicate that although LLMs such as Claude 3.5 Sonnet have potential for scientific tasks, other models may require more development or additional prompt engineering to reach comparable accuracy. Reviewers’ perceptions of artificial intelligence (AI) utility and trustworthiness showed a positive shift after evaluation. However, ethical concerns, particularly with respect to transparency and disclosure, remained consistent.

**Discussion:**

The study highlights the need for structured frameworks for evaluating LLMs and ethical considerations essential for responsible AI integration in scientific research. These findings should be interpreted with caution, as the limited sample size and domain-specific focus of the exam questions restrict the generalizability of the results.

## Graphical abstract

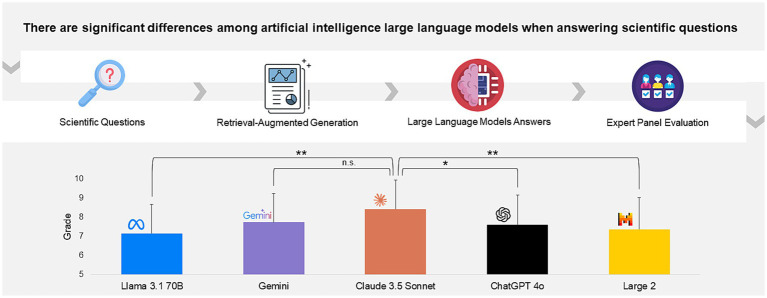

## 1 Introduction

Rapid advancements in artificial intelligence (AI), particularly in large language models (LLMs), are transforming the landscape of scientific research by streamlining knowledge discovery, data analysis, and hypothesis generation (Borger et al., 2023). LLMs are highly sophisticated models trained on vast and diverse text corpora, with architectures that enable them to process and generate human-like language (Rudolph et al., 2023). Recent developments have produced state-of-the-art models capable of deriving insights from complex, domain-specific data with remarkable depth and accuracy (Liu et al., 2024). However, while their potential in scientific applications is evident, a critical question remains: how do these models compare in their ability to accurately retrieve, interpret, and synthesize specialized information across different fields? Addressing this question is essential for understanding their reliability as tools for scientific inquiries.

Several recent studies have attempted to benchmark LLMs in scientific or technical contexts, often focusing on single models or narrow applications such as medical question answering, patient communication, or domain-specific tasks (Shool et al., 2023; Jahan et al., 2024; Li et al., 2024; Wang et al., 2024). These contributions provide valuable insights but generally lack systematic cross-model comparison combined with domain-specific retrieval augmentation and expert-based evaluation. By integrating these components, our study addresses this gap and proposes a reproducible benchmarking framework that expands beyond existing work.

This study compares leading LLMs systematically to determine which LLMs are best suited to answer complex scientific questions. By assessing the depth, accuracy, relevance, and clarity of each model, we establish a benchmark for its effectiveness in scientific research, revealing both its current strengths and limitations. Given the diverse architectures, training data, and design approaches across LLMs, this comparative analysis offers valuable insights for researchers and practitioners in various fields. A secondary aim of this study is to develop an optimized workflow to improve LLM performance in scientific contexts via retrieval-augmented generation (RAG) (Li and Ouyang, 2024) techniques for improved prompt generation. By expanding initial queries with targeted domain-specific information, RAG enables models to retrieve more relevant data. Our methodology, implemented through Jupyter notebooks (fully and freely available), refines question prompts with specific scientific literature, increasing the precision of LLM outputs and offering a practical framework for researchers to maximize these tools in their work.

This research contributes to the growing integration of AI in scientific inquiry and focuses on identifying which LLMs are best equipped for the complex interdisciplinary synthesis needed to drive scientific discovery. Our work stands out by employing a novel, objective, and quantifiable methodology that enables reliable comparisons of LLMs. The insights gained in this study will guide researchers in effectively leveraging AI for their needs, identify the strengths and limitations of prominent LLMs, and help chart future improvements toward a more reliable and powerful role for AI in scientific research.

## 2 Materials and methods

### 2.1 Selection of LLMs

Five state-of-the-art LLMs were selected for this study on the basis of their prominence and broad availability. The most advanced open-access LLMs available were used during August 2024. These models included Claude 3.5 Sonnet (Anthropic), Gemini (Google), GPT-4o (Open AI), Large 2 (Mistral AI) and Llama 3.1 70B (Meta). Each LLM was tested for its ability to answer a set of complex scientific questions pertaining to the extraction of bioactive compounds from agricultural byproducts.

### 2.2 Question set design

An exam composed of 10 scientifically rigorous questions was designed by the authors to assess the ability of each model to understand and synthesize knowledge. The questions listed below cover a wide range of key topics in the fields of agricultural byproducts, green extraction and sustainability:

1. What are agricultural byproducts, and why are they significant in the context of compound extraction?
2. What are the main advantages of using agricultural byproducts for compound extraction compared with traditional raw materials?
3. Describe the general process of extracting and/or purifying bioactive compounds from agricultural byproducts.
4. Explain the role of solvents in the extraction process and name at least three commonly used. Include at least one solvent recognized as novel or green.
5. Identify and describe three common methods used for extracting compounds from agricultural byproducts.
6. Discuss the environmental and economic impact of extracting compounds from agricultural byproducts.
7. What are some of the challenges associated with the extraction of compounds from agricultural byproducts?
8. Explain how the quality and composition of agricultural byproducts can affect the extraction process and yield of compounds.
9. Describe a case study in which a specific compound was successfully extracted from an agricultural byproduct.
10. What future trends or advancements do you foresee in the field of compound extraction from agricultural byproducts?

### 2.3 Retrieval-augmented generation (RAG) strategy

To obtain reliable contextual scientific information, a search using the terms “Extraction AND Agricultural AND Byproduct” was performed in the Scopus database on July 12, 2024, and 306 articles were obtained. The information from these articles, including their abstracts, was downloaded in a CSV file. The information from this file was used by a Jupyter notebook in Google Colab to serve as context for the scientific questions in the exams. Before the questions were answered, each LLM was tasked with performing a query expansion to refine the search and retrieval of scientific abstracts used as context before answer generation. The expanded queries allowed the models to select a more targeted set of relevant documents from scientific databases, thereby increasing the relevance and accuracy of their answers. By using embeddings after the query expansion was performed using each of the different LLMs, the article abstracts were selected to answer each of the questions. Using this information, “superprompts” were generated, including the specific context, the scientific question and clear instructions to answer each of the questions on each exam with each LLM. A complete set of prompts, questions, answers, databases, notebooks, and code is openly available in the OSF repository (see Data Availability Statement).

### 2.4 Answer generation

**Figure 1:**
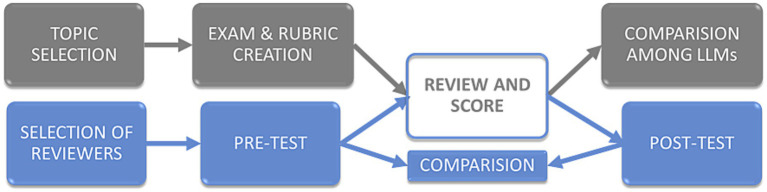
Study workflow. Gray boxes represent the stages of exam creation and evaluation, while blue boxes depict the steps undertaken by the co-authors serving as reviewers. The “Review and Score” box, colored both gray and blue, signifies its inclusion in both pipelines.

Once the query expansion and retrieval were complete, each LLM was instructed to answer the 10 exam questions one by one on the basis of the generated “superprompts.” Each question was asked with its corresponding “superprompt” in a new chat to minimize possible interference. The answers were anonymized and formatted uniformly for subsequent evaluation by human experts. Exams were coded as Exam 1, Exam 2, Exam 3, Exam 4 and Exam 5. Only the first author of this work, who did not evaluate any exams, knew which LLM had solved each exam. The order of the exams (1 to 5) was chosen by random number generation. A diagram of the process from obtaining the articles for the scientific context to obtaining the answers for each LLM is shown in Figure 1. All LLMs were accessed through their free public interfaces, which did not allow modification of hyper-parameters such as temperature or maximum tokens. As a result, all experiments were performed using the default configurations of each platform, which we recognize as a methodological limitation.

### 2.5 Expert evaluation panel

A panel of 16 human experts from 7 different countries and 14 different institutions (Supplementary Table 1) was assembled to evaluate the answers generated by the LLMs. These experts were selected on the basis of their research expertise in fields related to agricultural byproduct valorization, bioactive compound extraction, and green chemistry. The panel members have h-indices between 10 and 58, with a mean value of 35.06 and a median of 38.

All responses were anonymized and uniformly formatted before scoring, and evaluators were blinded to which LLM had generated each set of answers. Only the non-evaluating authors, who designed the exam and prompts but did not participate in the evaluation, knew the assignment of models to exams. A standardized rubric defining the criteria of accuracy, depth, relevance, and clarity was provided to all evaluators in advance. No formal calibration sessions were conducted, as the aim was to preserve the evaluators’ natural heterogeneity and thereby reflect the diversity of perspectives present in the broader scientific community. The panel itself was diverse in nationality, institutional background, age, gender and experience, which further strengthens the representativeness of the evaluation.

### 2.6 Evaluation criteria

The anonymized answers from each LLM were scored by the panel using a scale of 0 to 10, with a single standardized rubric containing the following evaluation criteria: accuracy, i.e., how correct and factually precise the answer is, consistent with the current state of scientific knowledge; depth, i.e., the level of detail and comprehensiveness provided in the explanation; relevance, i.e., how well the answer addressed the specific question; and clarity, i.e., the ease of understanding and logical structure of the response. The rubric was co-designed by the authors to ensure consistency across evaluations, with clear definitions for accuracy, depth, relevance, and clarity provided to all reviewers in advance. Each reviewer applied the same standardized criteria independently. Statistical analysis was performed using the Friedman test, which is appropriate for non-parametric repeated measures (Shi et al., 2024), to account for the fact that all LLMs were evaluated by the same panel of experts. This approach ensured methodological rigor despite the limited sample size.

### 2.7 Pre- and postevaluation questionnaires

To gather additional insights into expert perceptions, two sets of questionnaires were administered. A preevaluation questionnaire in which experts were asked about their familiarity with AI and LLMs, their expectations for AI performance in scientific applications, and any preconceptions they might have regarding the utility of AI in research. A postevaluation questionnaire in which, after reviewing the AI-generated answers, experts were surveyed on their perceptions of the quality of the responses and their views on the potential for LLMs to assist in scientific research.

### 2.8 Statistical analysis

The scores from the expert evaluations were compiled and averaged for each LLM. The results were then analyzed using the Friedman test to identify any significant differences in performance across the LLMs.

## 3 Results

To ensure a rigorous and precise evaluation of the various LLMs, a topic within the authors’ expertise was selected. An exam comprising 10 questions (see the Supplementary material) and a rubric for assessment were subsequently developed (gray boxes in Figure 1). Concurrently (blue boxes in Figure 1), a panel of 16 coauthors was assembled, and an initial survey (pretest) was administered to gage their prior experience with AI and LLM models. These panelists then proceeded to evaluate and score the responses generated by the different LLMs. After the evaluation, the coauthors who served as reviewers completed a second survey (posttest) that reflects their experiences. A comparative analysis was conducted between the scores and responses of the LLMs and the reviewers’ answers before and after the evaluation.

### 3.1 LLMs expert panel comparison

After the review and scoring process conducted by the panel of reviewers, the average scores and standard deviations achieved by each LLM were analyzed to assess their efficacy in generating scientifically accurate and relevant responses. All coauthors who served as reviewers evaluated the responses using a blind approach to mitigate any potential bias stemming from their prior experience or knowledge of LLMs.

To promote open access science, the five most prominent and free-access models were tested: Llama 3.1 70B, Gemini, Claude 3.5 Sonnet, ChatGPT 4o, and Mistral Large 2. Each model was blindly evaluated by a panel of 16 experts on the basis of its ability to provide accurate, comprehensive answers to an exam composed of a set of complex scientific questions regarding green chemistry and sustainability.

Claude 3.5 Sonnet achieved the highest average score, with a mean rating of 8.41 ± 1.53 (out of 10). Gemini followed with a mean score of 7.73 ± 1.50, reflecting a solid performance with relatively low variability. ChatGPT 4o also demonstrated competitive results, with an average score of 7.60 ± 1.55, showing its ability to produce reliable responses across various queries. Llama 3.1 70B and Mistral Large 2 received slightly lower average scores of 7.36 ± 1.51 and 7.15 ± 1.66, respectively. Mistral Large 2 had the highest standard deviation, which indicates slightly greater fluctuation in performance. There were significant differences among the grades obtained by the different LLMs (Table 1). Claude 3.5 Sonnet had a significantly better average grade than Llama 3.1 70 B (*p* < 0.01), Mistral Large 2 (*p* < 0.01) and ChatGPT 4o (*p* < 0.05); however, the average grade was not significantly different from that of Gemini (*p* = 0.38). A heatmap with comparisons of the statistical significance among the grades obtained by each LLM is shown in Supplementary Figure 1.

**Table 1:** Average grades and standard deviations obtained by each LLM after evaluation by expert scientific reviewers.

LLM	Mean	Standard deviation
Llama 3.1 70B	7.15^b^	1.51
Gemini	7.73^a,b^	1.50
Claude 3.5 Sonnet	8.41^a^	1.53
ChatGPT 4o	7.60^b^	1.55
Mistral Large 2	7.36^b^	1.66

Different letters (a, b) indicate significant differences among LLMs on the basis of the Friedman test (*p* < 0.05).

These findings suggest that, while all the tested LLMs demonstrated competence in addressing scientific queries, Claude 3.5 Sonnet and Gemini outperformed the other models in terms of overall accuracy and reliability, with Claude 3.5 Sonnet emerging as the top performer. This comparative analysis provides valuable insights into the strengths and limitations of each model and guides researchers in selecting the most suitable LLM for applications that demand high-quality scientific outputs.

**Figure 2:**
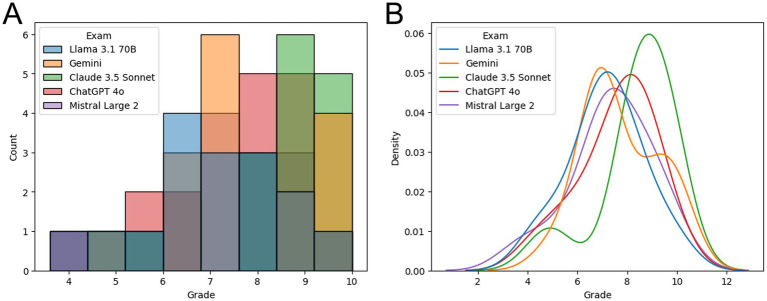
**(A)** Histogram showing the reviewers’ grades for the exam conducted by each LLM. **(B)** Kernel density estimate plot for LLM grades probability density.

To improve visualization of the differences among the LLMs, the results were represented as a histogram (Figure 2A) and as a kernel density estimate plot for continuous probability density (Figure 2B). Claude 3.5 Sonnet (in green) stands out from the other four LLMs.

**Figure 3:**
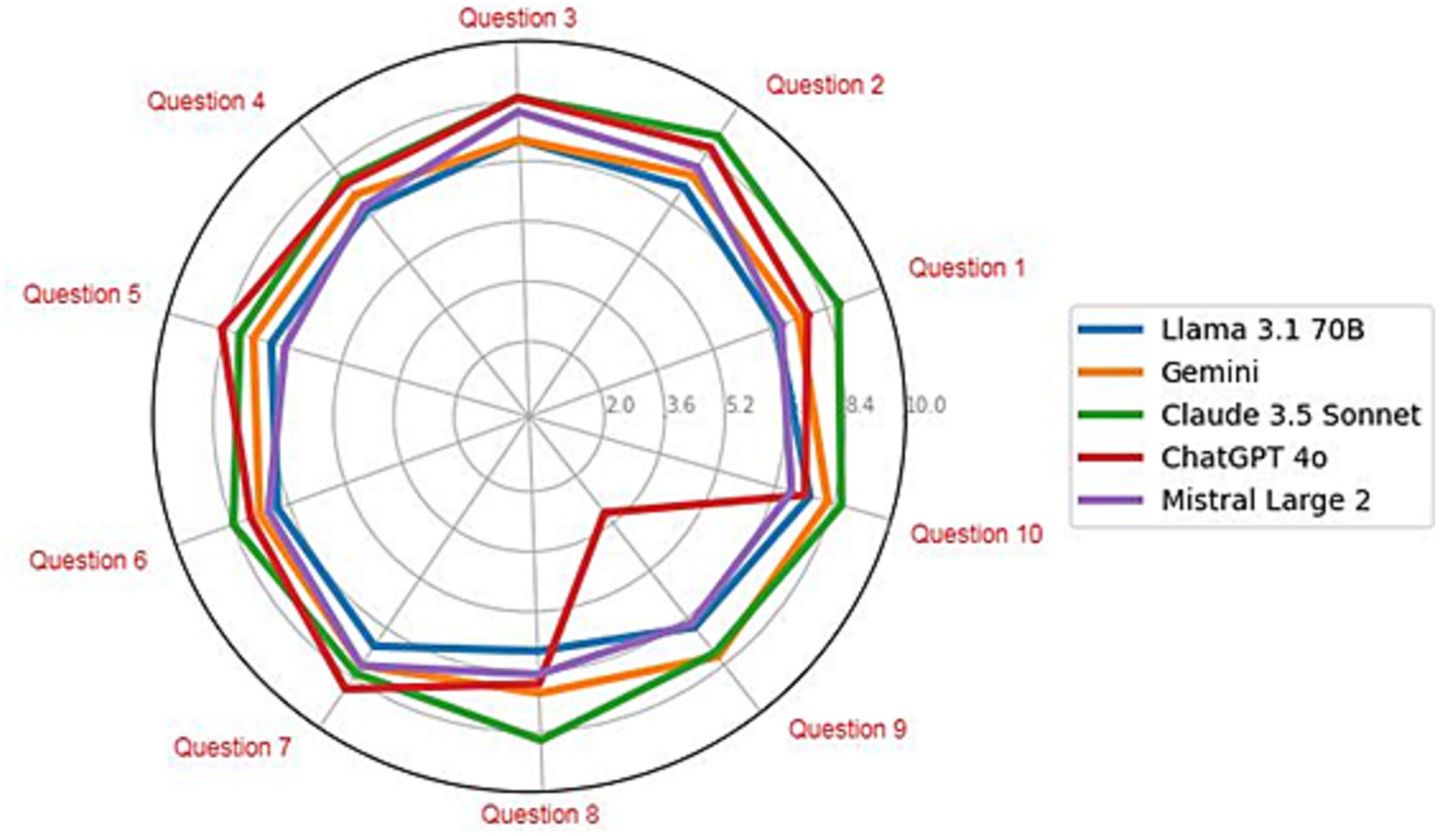
Radial chart with the average scores of each LLM for each specific question in the exams evaluated by the panel of reviewers.

The average scores obtained for each specific exam question for each LLM were analyzed (Figure 3). All the models exhibited a circular pattern, which suggests consistent performance across all the questions, with minor variations affecting the final score. However, a drastic decrease in the ChatGPT 4o grade can be clearly observed in question 9: “Describe a case study where a specific compound was successfully extracted from an agricultural byproduct.” In this case, the LLM focused on summarizing key points from the related literature but failed to provide a concrete example as it was requested. On the other hand, the superior performance of Claude 3.5 Sonnet on question 8, “Explain how the quality and composition of agricultural byproducts can affect the extraction process and the yield of compounds,” likely results from its ability to analyze and contextualize scientific information in detail. This model has excelled in identifying and articulating the specific relationships between the quality and composition of agricultural byproducts and their influence on extraction efficiency and yield. Its approach might reflect better integration of key concepts, a clearer structure in presenting cause–effect relationships, and a focus on critical variables, resulting in a more insightful and precise answer.

### 3.2 Evaluation questionnaires

In order to complement the performance evaluation of the LLMs, we also analyzed how reviewers’ perceptions of AI evolved before and after the assessment. While methodologically distinct, these two components are presented together in order to capture not only how the models performed but also how their use was perceived by domain experts.

To assess the evaluators’ baseline perspectives on AI tools before the LLM-generated scientific responses were reviewed, a preevaluation questionnaire was administered to the 16 reviewers. The questionnaire included five questions concerning their current AI usage, future intentions to use AI, perceived usefulness, trustworthiness, and views on ethical considerations. The responses provided insight into the prevailing attitudes toward AI applications in scientific research. After the LLM-generated responses were evaluated, a postevaluation questionnaire was administered to the same 16 reviewers. These questions were aimed at capturing shifts in perspectives, which enabled a comparison with the preevaluation questionnaire results and offered insight into whether and how direct engagement with AI-involved tasks influenced their views.

**Figure 4:**
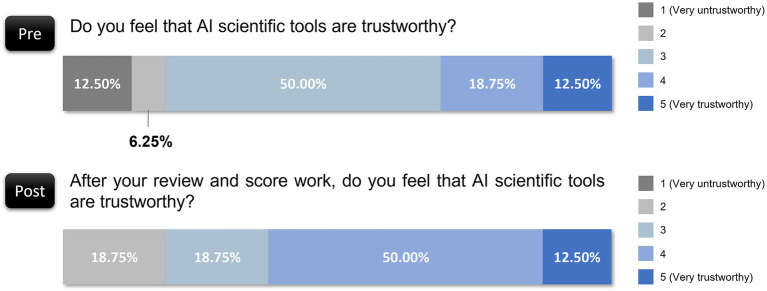
Shift in AI tools adoption intentions among reviewers before and after evaluation.

In the preevaluation questionnaire, the reviewers expressed mixed intentions regarding short-term AI use: 37.50% were unsure, 25.00% intended to start using AI, and 37.50% were already using AI. Postevaluation, intentions leaned toward greater adoption. Only 18.75% were still uncertain, and the proportion of those already using AI or planning to increase their AI usage increased to 81.25%. Notably, 31.25% indicated that they would use more AI tools than they currently do, which reflected a significant postevaluation shift toward the integration of AI in daily scientific work (Figure 4). This finding suggests that exposure to AI’s potential through the evaluation task heightened awareness of its applicability, fostering a positive shift in adoption intentions.

**Figure 5:**
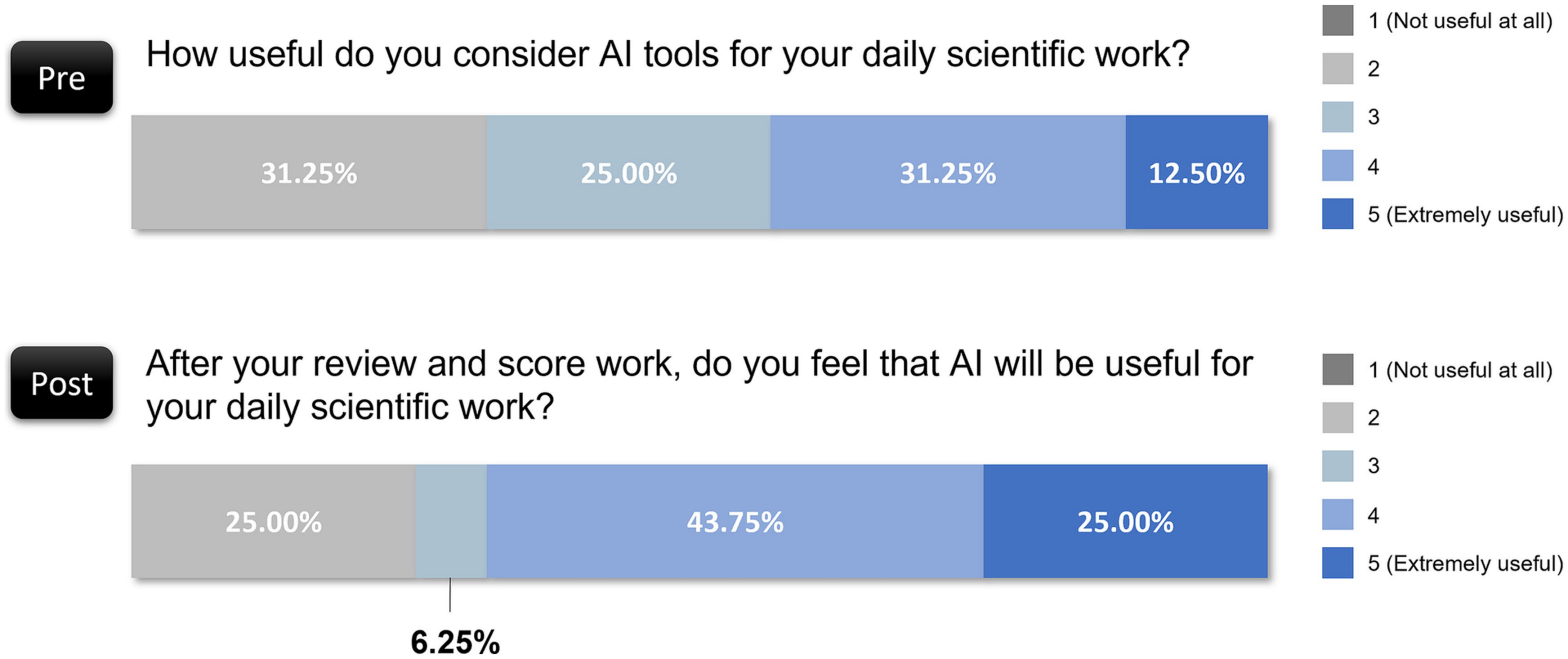
Changes in reviewers’ perceptions of AI utility before and after evaluation.

The preevaluation survey revealed that 31.25% of the respondents rated AI as being minimally useful (rating of 2 out of 5), and only 12.50% considered it extremely useful (rating of 5 out of 5). After the evaluation task, perceptions of the utility of AI improved: 43.75% rated it moderately useful (rating of 4 out of 5), and another 25.00% found it extremely useful (rating of 5 out of 5). This upward trend signifies that interaction with AI-based models highlighted tangible benefits for some reviewers, who became more convinced of its relevance to scientific tasks (Figure 5), despite the remaining skepticism among 25.00% who still rated the usefulness of AI at a low level (rating of 2 out of 5).

Half of the reviewers (50.00%) reported a moderate shift in their views (rating 3 out of 5) postevaluation, whereas only 18.75% claimed to have experienced no change in perspective (rating of 1 out of 5). Moreover, 6.25% noted a significant positive shift (rating of 5 out of 5). The majority noted some level of change, which indicates that hands-on experience with AI output influenced perceptions, even if most adjustments were modest. The results suggested that direct engagement with AI may gradually influence scientific opinions, which encourages openness to AI integration over time.

**Figure 6:**
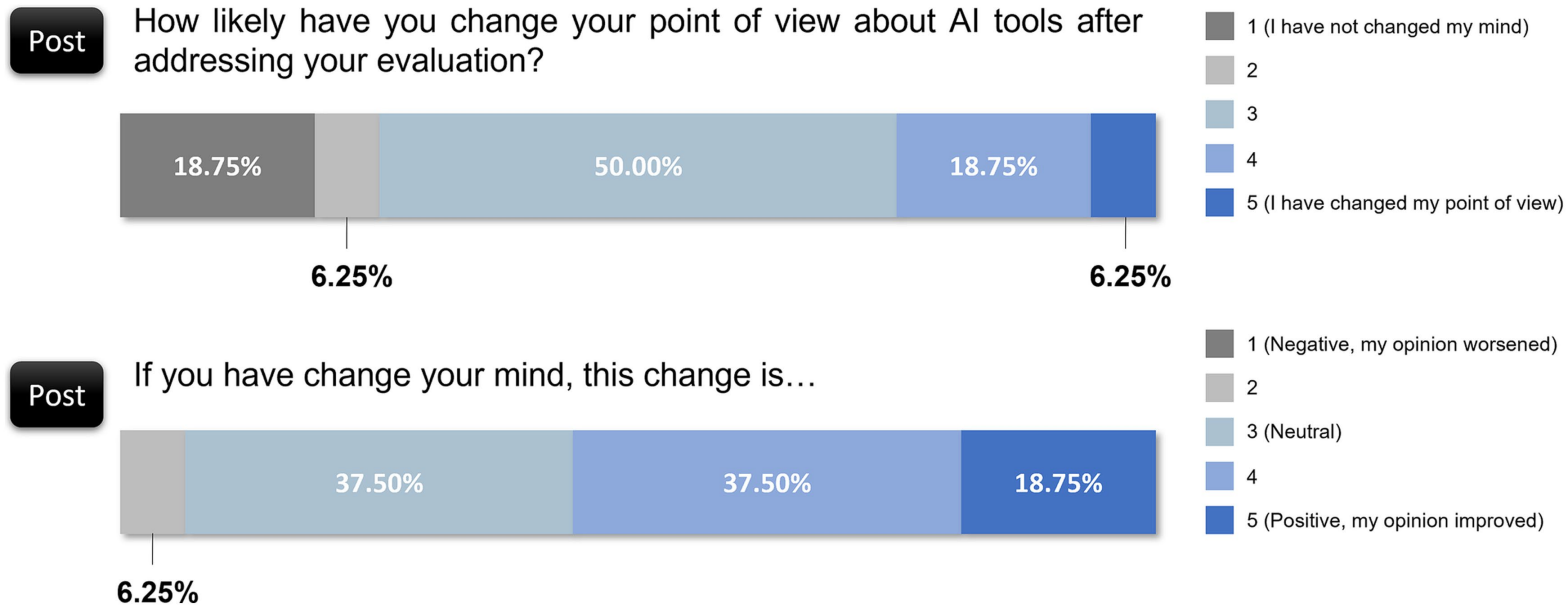
Shifts in reviewers’ perspectives on AI following the evaluation.

For those whose opinions shifted, the change tended to be positive or neutral. Specifically, 37.50% expressed a neutral stance (rating of 3 out of 5), 37.50% viewed AI more favorably postevaluation (rating of 4 out of 5), and 18.75% noted a distinct positive shift (rating of 5 out of 5). Only one reviewer (6.25%) reported a slightly negative change (Figure 6). This distribution indicates that interaction with AI tools in a controlled scientific context generally supports positive adjustments in perception, which potentially alleviates initial doubts or misconceptions.

**Figure 7:**
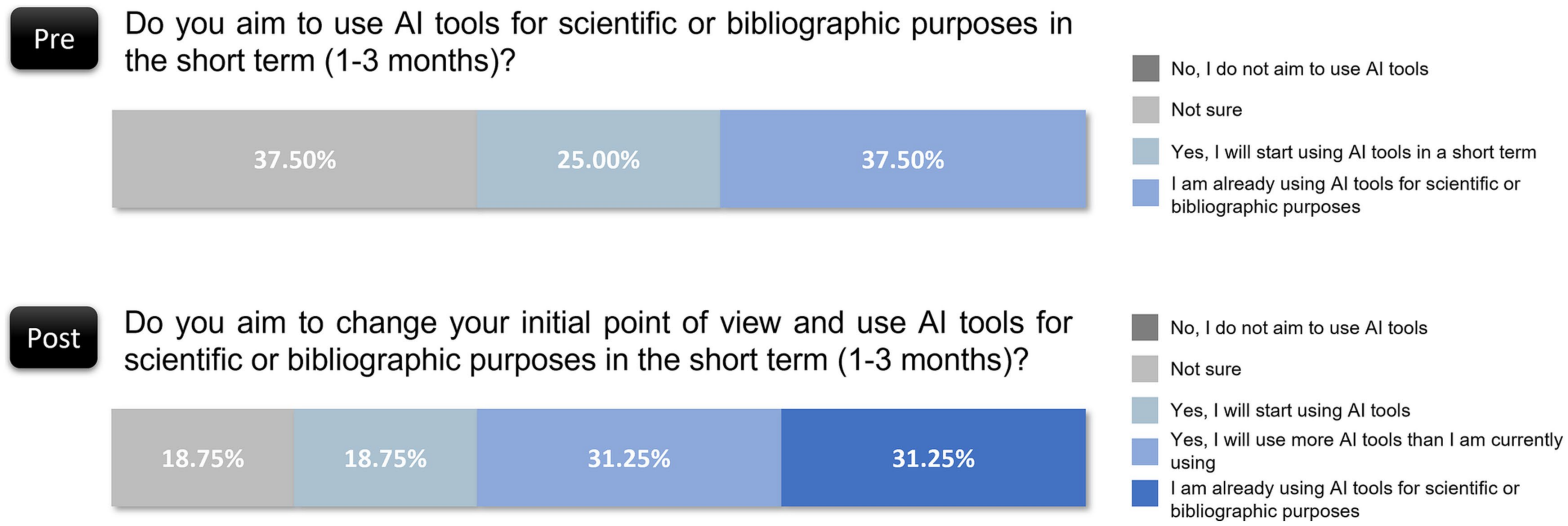
Changes in reviewers’ trust in AI tools before and after evaluation.

In terms of trust, the preevaluation responses were mixed, with only 18.75% of the respondents rating AI tools as highly trustworthy (rating of 4 or 5 out of 5). After the evaluation exercise, however, the trust ratings improved (Figure 7): 50.00% of the participants rated AI as fairly trustworthy (rating of 4 out of 5), and 12.50% rated it as very trustworthy (rating of 5 out of 5). These shifts indicate that reviewers became more comfortable with the reliability of AI tools after they observed their capabilities firsthand, although 18.75% still expressed low levels of trust (rating of 2 or 3 out of 5). This result points to a gradual building of confidence that may increase with further familiarity and reliability demonstrations.

**Figure 8:**
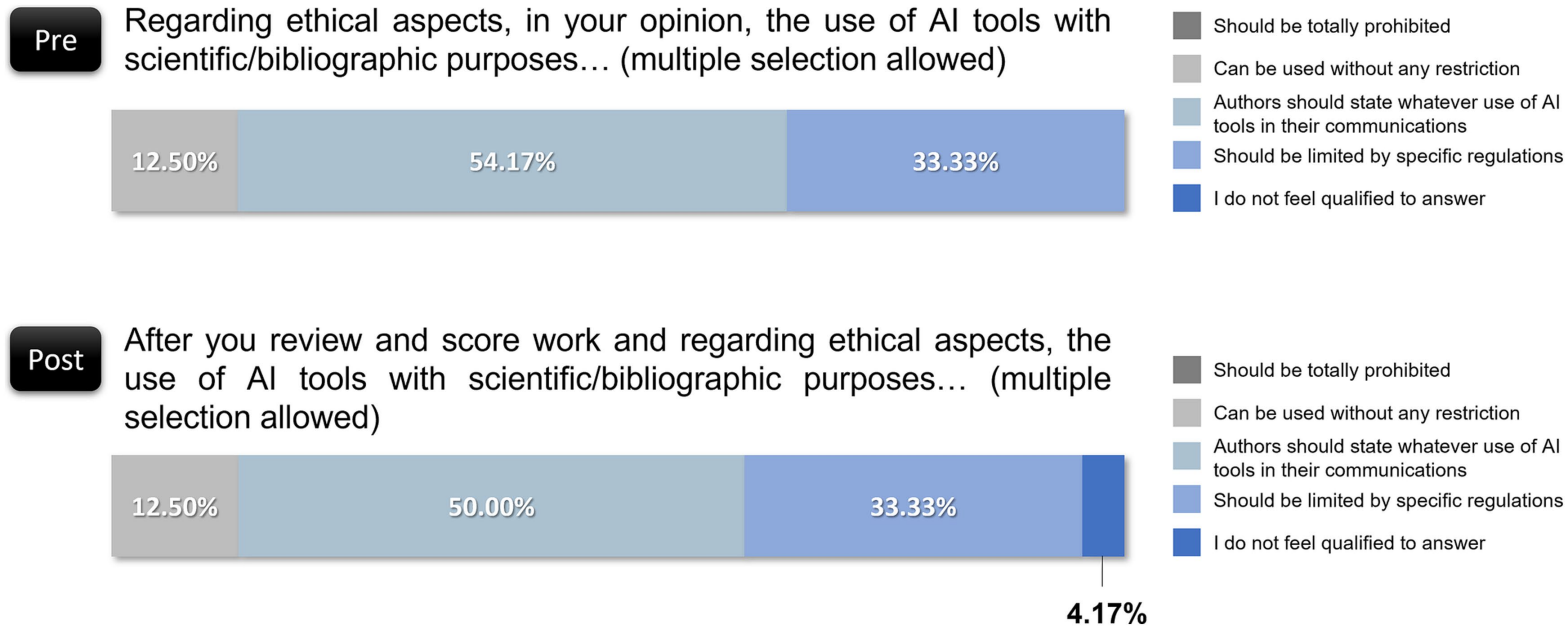
Reviewers’ perspectives on ethical considerations for AI usage before and after evaluating.

When revisiting ethical considerations, reviewers largely upheld the need for AI usage disclosure, with 50.00% agreeing that authors should specify any AI usage in scientific communications, a percentage that is very close to the preevaluation percentage (54.17%). Moreover, 33.33% supported regulations on AI use, a perspective that remained stable compared with preevaluation views. Notably, 12.50% endorsed unrestricted AI usage, identical to initial responses, and one reviewer (4.17%) expressed uncertainty. This finding suggests that while positive shifts occurred regarding AI utility and trustworthiness, ethical concerns remained steady, with most reviewers advocating for transparency and regulatory frameworks (Figure 8).

## 4 Discussion

**Figure 9:**
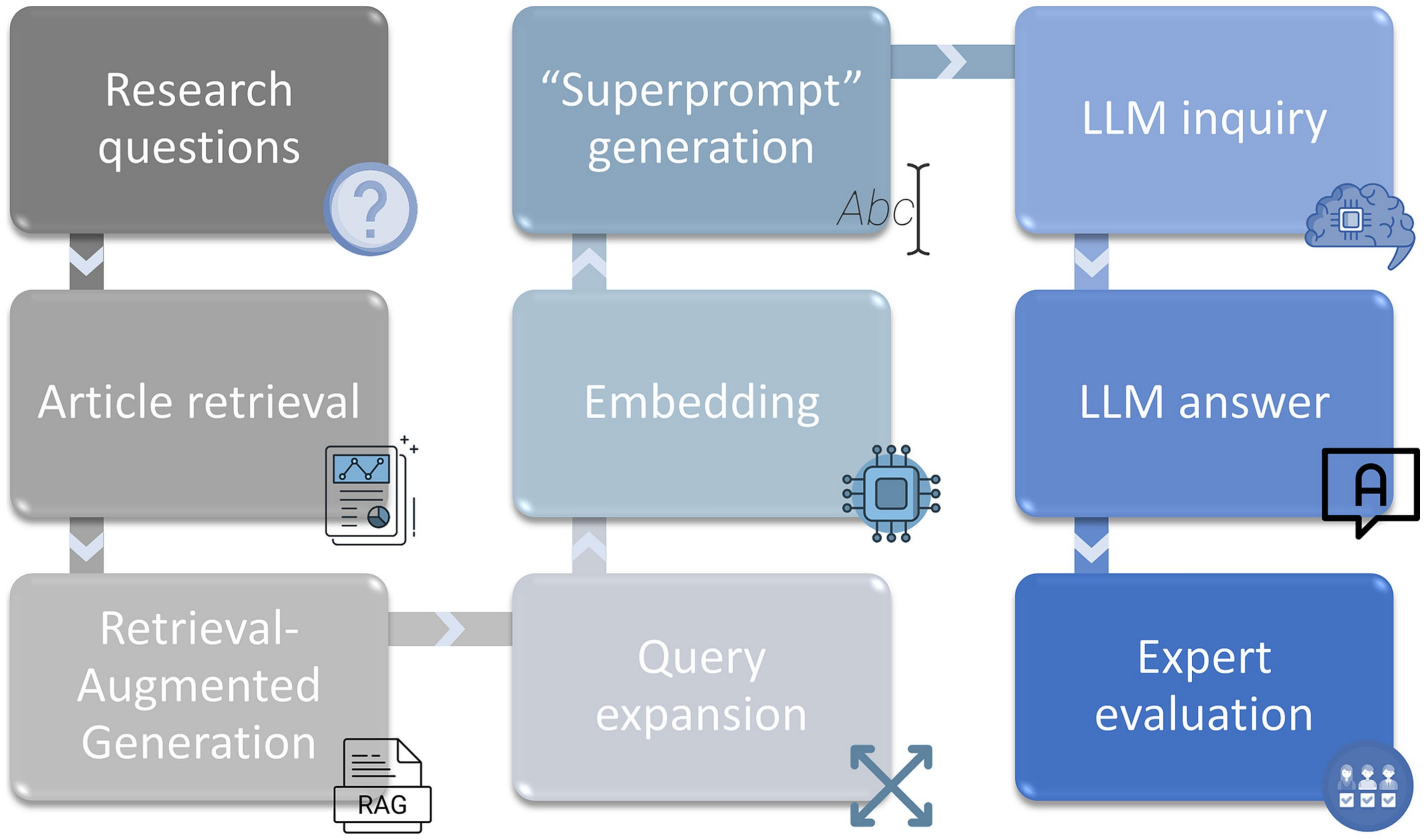
Diagram of the process of preparing information and answering each of the exam questions.

This study presents a novel contribution by rigorously comparing the performance of the most popular free LLMs in responding to complex scientific questions, as assessed by a diverse panel of international experts. This approach has not been extensively applied, which positions this work as an important benchmark for assessing LLM effectiveness (Figure 9).

A key limitation of this study is the limited sample size of exam questions, which inevitably constrains the scope of the conclusions. Although Claude 3.5 Sonnet achieved the highest score in our evaluation, this result should not be assumed to hold across other domains or scientific contexts. Indeed, our own findings support the reviewer’s observation that LLM performance can vary substantially depending on the subject area. For this reason, the outcomes of the present work should be regarded as preliminary and domain-specific. Future studies should increase both the number of questions and the diversity of disciplines in order to provide more robust validation.

Previous research has shown that LLMs can produce informative and often high-quality content across medical and health-related topics; however, these studies have typically focused on single LLMs or specific applications, such as generating patient information leaflets or assisting with medical decision-making (Pompili et al., 2024; Saibene et al., 2024; Sharifi et al., 2024; Goel et al., 2023). In a similar vein, recent studies have highlighted the potential of LLMs for performing complex tasks in medicine, such as health coaching and monitoring of chemotherapy-induced toxicity (Saibene et al., 2024; Ruiz Sarrias et al., 2024). However, these studies often rely on specific question types or isolated case studies, with limited cross-comparisons among LLMs. In contrast, our study addresses a broader spectrum of scientific queries in the context of food technology, agricultural byproducts, green extraction and sustainability, spanning different levels of complexity and contextual depth, which are fundamental to evaluating robustness of each model in real-world scientific applications.

A key strength of this study is its methodological design, which uses a RAG technique to optimize query relevance, a method that has been shown to increase LLM outputs by incorporating domain-specific knowledge (Cheng et al., 2023; Deiner et al., 2024). This augmentation aligns with recent calls in the literature for integrating specialized data sources to improve LLM performance on expert-level tasks, particularly in areas such as oncology and mental health, where nuanced information is essential (Ruiz Sarrias et al., 2024; Elyoseph and Levkovich, 2024). Additionally, our inclusion of a large and diverse expert panel for response evaluation adds depth to our findings. Unlike studies that rely solely on quantitative metrics, our approach captures qualitative insights regarding response accuracy, readability, and helpfulness—dimensions that are critical for scientific applicability but are often underexplored (Pompili et al., 2024; Kim and Kim, 2024).

Previous works have highlighted certain limitations in LLM-generated responses, such as difficulty in conveying specific case examples or maintaining accuracy in highly specialized topics (Mu et al., 2024; Kostic et al., 2024; Chen, 2024). Our results corroborate these findings, especially for ChatGPT 4o, which underperformed on a question requiring detailed case-based reasoning. This outcome suggests that LLMs, while valuable, may still struggle with domain-specific precision unless complemented by further prompt optimization or RAG techniques. Nonetheless, the high performance of Claude 3.5 Sonnet in delivering accurate and contextually relevant responses points to the potential of next-generation LLMs to meet the demands of complex scientific discourse, potentially paving the way for AI to become a reliable collaborator in scientific research (Deiner et al., 2024).

In this study we chose to report and compare the average scores assigned to each LLM by the expert panel. This approach was selected as it provides an intuitive and interpretable measure of overall performance, while also allowing statistical comparison across models. We acknowledge that complementary descriptors, such as medians or distributions of scores, could provide additional insights and should be considered in future studies.

An important methodological consideration is that all evaluators were also co-authors. However, we do not regard this as a conflict of interest, since the evaluation process was fully blinded: none of the evaluators knew which LLM generated each answer, and only the first authors who did not serve as evaluators had access to this information. Several safeguards were in place, including anonymization of all responses and independent scoring without discussion among panel members. Furthermore, while all evaluators followed the same standardized rubric, no calibration beyond this rubric was imposed, in order to reflect the heterogeneity of scientific judgment. This approach, combined with the diversity of our panel (in terms of country, institution, age, and gender), ensures that the results reflect a realistic range of expert perspectives rather than artificially homogenized assessments.

By positioning this study within the broader landscape of LLM research, our findings provide a comprehensive view of current LLM capabilities and highlight the importance of continuous model refinement. Future research should build on this approach by exploring more interactive and adaptive models, particularly those capable of real-time adjustments based on human feedback, to better meet the evolving needs of scientific inquiry (Cheng et al., 2023; Kim and Kim, 2024). As AI continues to integrate into research workflows, establishing reliable benchmarks for LLM performance and ethical guidelines for their use will be essential in safeguarding the quality and integrity of scientific output (Elyoseph and Levkovich, 2024).

While the exploratory nature and domain specificity of this study may resemble certain aspects of a community case study, we believe the systematic benchmarking, statistical analysis, and integration of expert perspectives clearly situate this work within the scope of original research. By combining quantitative evaluation with qualitative insights, the study provides a rigorous and reproducible framework that contributes to the broader field of LLM assessment in scientific contexts.

The performance disparities among LLMs, as evidenced in our comparative analysis, highlight a critical issue: inconsistencies in AI-generated outputs can result in divergent interpretations when LLMs are relied upon for scientific purposes (Qu and Wang, 2024; Wu et al., 2024). This variability not only affects the perceived reliability of LLMs but also raises questions regarding their interpretability and transparency in research contexts. Our results stress the need for transparency and explainability in AI tools used in scientific research, because these factors are essential for building trust among researchers and maintaining rigorous scientific standards.

The ethical considerations expressed by our panel of reviewers both pre- and postevaluation emphasize the importance of disclosure and regulatory oversight when LLMs are integrated into research workflows. Transparency regarding AI involvement in scientific analysis, as supported by more than half of our reviewers, is crucial for preserving research integrity and mitigating potential biases introduced by AI systems. These findings resonate with the growing consensus in the AI ethics literature, which advocates for disclosure practices that inform readers of AI contributions (Perkins, 2023; Hosseini et al., 2023; Kiseleva et al., 2022; Chen et al., 2022), thereby fostering accountability and trust.

The shift in reviewer perspectives after direct interaction with LLM-generated outputs suggests that familiarity and hands-on experience with AI tools may alleviate initial skepticism. Nonetheless, the consistent emphasis on ethical disclosure implies a foundational need for clear regulatory guidelines to govern AI use in scientific research. Establishing these frameworks would provide researchers with a structured pathway for ethically incorporating AI, thereby aligning with the broader goals of transparency, reliability, and societal trust in AI-driven research.

Future research should expand this benchmarking approach by incorporating a broader array of LLMs and scientific disciplines, thereby providing a comprehensive understanding of LLM utility across fields. Additionally, advancements in LLM interpretability and transparency tools, such as model auditing and explainable AI (XAI) techniques (Ali et al., 2023), could address several ethical concerns highlighted by our reviewers. These innovations would support the development of LLMs capable of not only generating accurate scientific responses but also providing researchers with insights into the underlying reasoning, ultimately advancing AI as a robust and trustworthy partner in scientific discovery.

## 5 Conclusion

This study illuminates both the potential and limitations of current LLMs in addressing intricate scientific inquiries. Claude 3.5 Sonnet stands out for its accuracy and consistency, which suggests that advancements in LLM architecture and specialized training can indeed produce tools that support and, in some cases, increase scientific work. However, our findings also highlight that, across the field, LLMs remain limited by inconsistencies and occasional gaps in domain-specific knowledge.

The significance of this research extends beyond the technical performance of LLMs: it calls for a critical approach to their integration into scientific workflows, demanding transparency, methodological rigor, and ethical foresight. As LLMs become increasingly common in research settings, understanding when and how they should be applied is essential to harness their strengths while mitigating their limitations. The ethical implications of AI in science, highlighted by our reviewers’ feedback, emphasize the need for explicit disclosure and regulation to maintain integrity and trust in AI-assisted findings.

Given the exploratory nature of this work and the limited number of exam questions, the conclusions should be considered preliminary. Larger and more diverse datasets, as well as expanded disciplinary coverage, will be necessary to confirm and extend these findings. Future work should also include replication with independent, blinded evaluators outside the authorship team, to confirm whether the comparative performance patterns observed here remain stable across panels.

## Data Availability

The datasets presented in this study can be found in online repositories. The names of the repository/repositories and accession number(s) can be found in the article/Supplementary material.
