# Supplementary material for: There are significant differences among artificial intelligence large language models when answering scientific questions
Source: Front Artif Intell. 2025 Oct 9;8:1664303. doi: 10.3389/frai.2025.1664303 (PMC12547693; doi:10.3389/frai.2025.1664303)
Supplement: Supplementary file 1 [file Data_Sheet_1.docx]

Supplementary Material

# Supplementary Figures and Tables

**
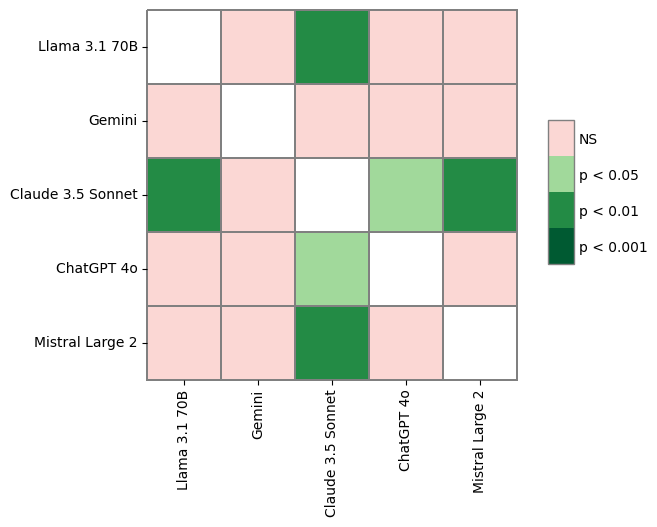
**

**Supplementary Figure 1.** Heatmap with comparisons between the statistical significance among the grades obtained by each LLM.

**Supplementary Table 1.** Expert panel key information.

| Evaluator | Country | Institution |
| --- | --- | --- |
| Dr. Luis Esteban | Spain | CIEMAT |
| Dr. Lucas Frungillo | England | University of Edinburgh |
| Dr. Estefanía Butassi | Argentina | CICONET |
| Dr. Alessandro Zambon | Italy | Università di Bologna |
| Dr. María Herranz | Spain | Miguel Hernández University |
| Dr. Mario Aranda | Chile | Universidad Católica de Chile |
| Dr. Federica Pollastro | Italy | Piamonte Orientale University |
| Dr. Anne Sylvie Tixier | France | Avignon University |
| Dr. Enrique Barrajón | Spain | Miguel Hernández University |
| Dr. Jose Vicente García | Spain | Polytechnic University of Valencia |
| Dr. David Arráez | Spain | Granada University |
| Dr. Andrew Ross | England | Leeds University |
| Dr. Pedro Mena | Italy | Parma University |
| Dr. Ru Angelie Edrada-Ebel | Scotland | University of Strarthclyde |
| Dr. James Lyng | Ireland | University College Dublin |
| Dr. Vicente Micol | Spain | Miguel Hernández University |
